# Supplementary material for: Erythropoietin decreases apoptosis and promotes Schwann cell repair and phagocytosis following nerve crush injury in mice
Source: Cell Death Dis. 2025 Jul 3;16(1):490. doi: 10.1038/s41419-025-07825-4 (PMC12229349; doi:10.1038/s41419-025-07825-4)
Supplement: Supplementary file 1 — Supplemental Material [file 41419_2025_7825_MOESM1_ESM.docx]

**SUPPLEMENTAL MATERIAL**

**Erythropoietin decreases apoptosis and promotes Schwann cell repair and phagocytosis following nerve crush injury in mice**

Prem Kumar Govindappa^1*^, Govindaraj Ellur^1^, John P. Hegarty^2^, Akash Gupta^3^, Rahul, V. G.^1^, and John C. Elfar^1*^

^1^Department of Orthopaedics and Sports Medicine, University of Arizona College of Medicine, Tucson, AZ, 85724, USA.

^2^Department of Cellular and Molecular Physiology, The Pennsylvania State University College of Medicine, Hershey, PA, 17033, USA.

^3^Department of Medicine, College of Medicine, University of Arizona, Tucson, AZ, 85724, USA.

*These authors share corresponding authorship.

**Correspondence:**

**Prem Kumar Govindappa**

email: [pkgovindappa@gmail.com](mailto:pkgovindappa@gmail.com)

**John C. Elfar**

email: [openelfar@gmail.com](mailto:openelfar@gmail.com)

**
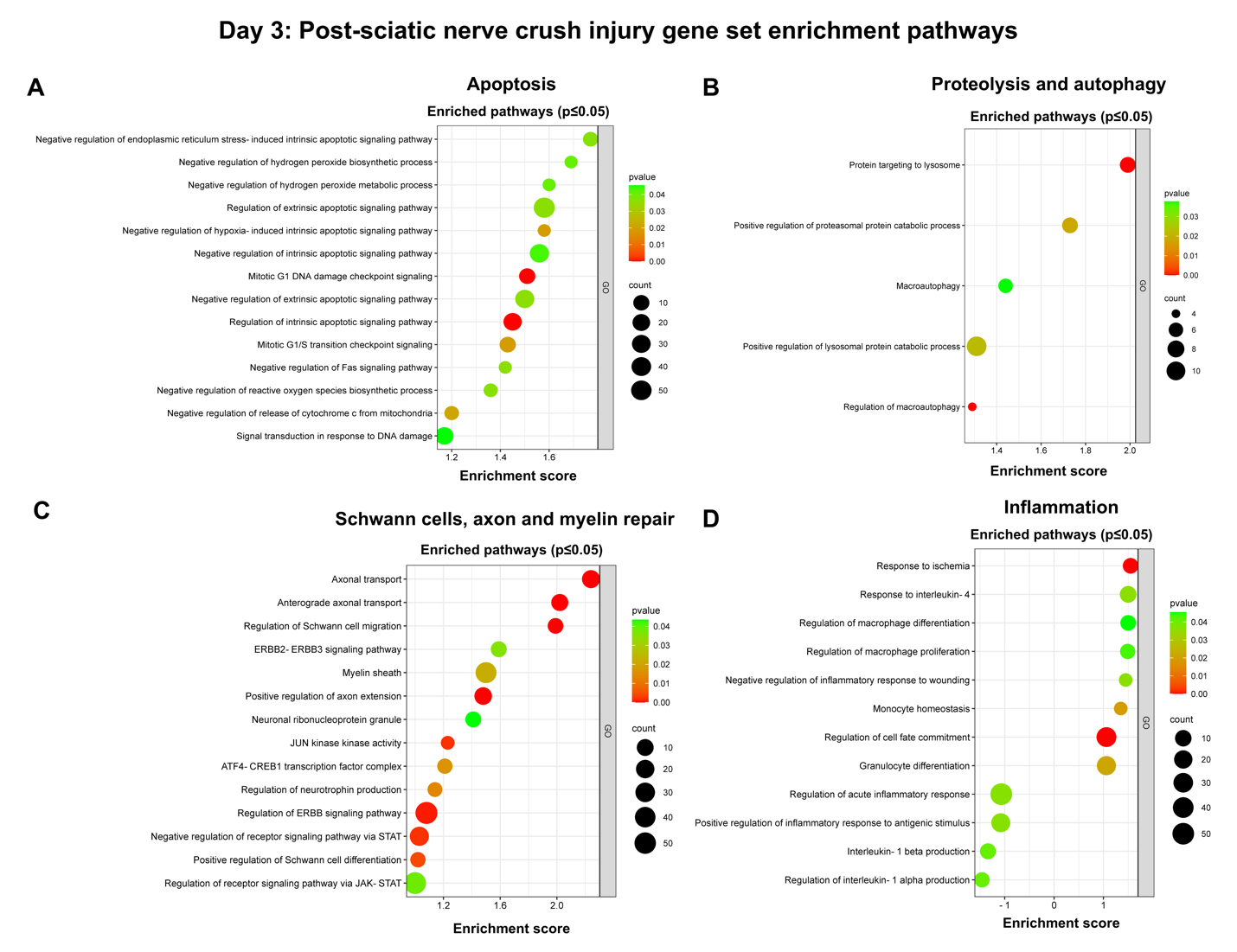
Supplementary Fig. 1: On Day 3, bulk RNA sequencing revealed EPO-enriched genes for biological pathways in nerves following SNCI. A-D** Gene set enrichment assay (GSEA) pathways are represented on the y-axis with their associated gene numbers (p ≤ 0.05), while the x-axis displays the enrichment score for each pathway. Saline vs. EPO treatment, n = 3/ group.

**
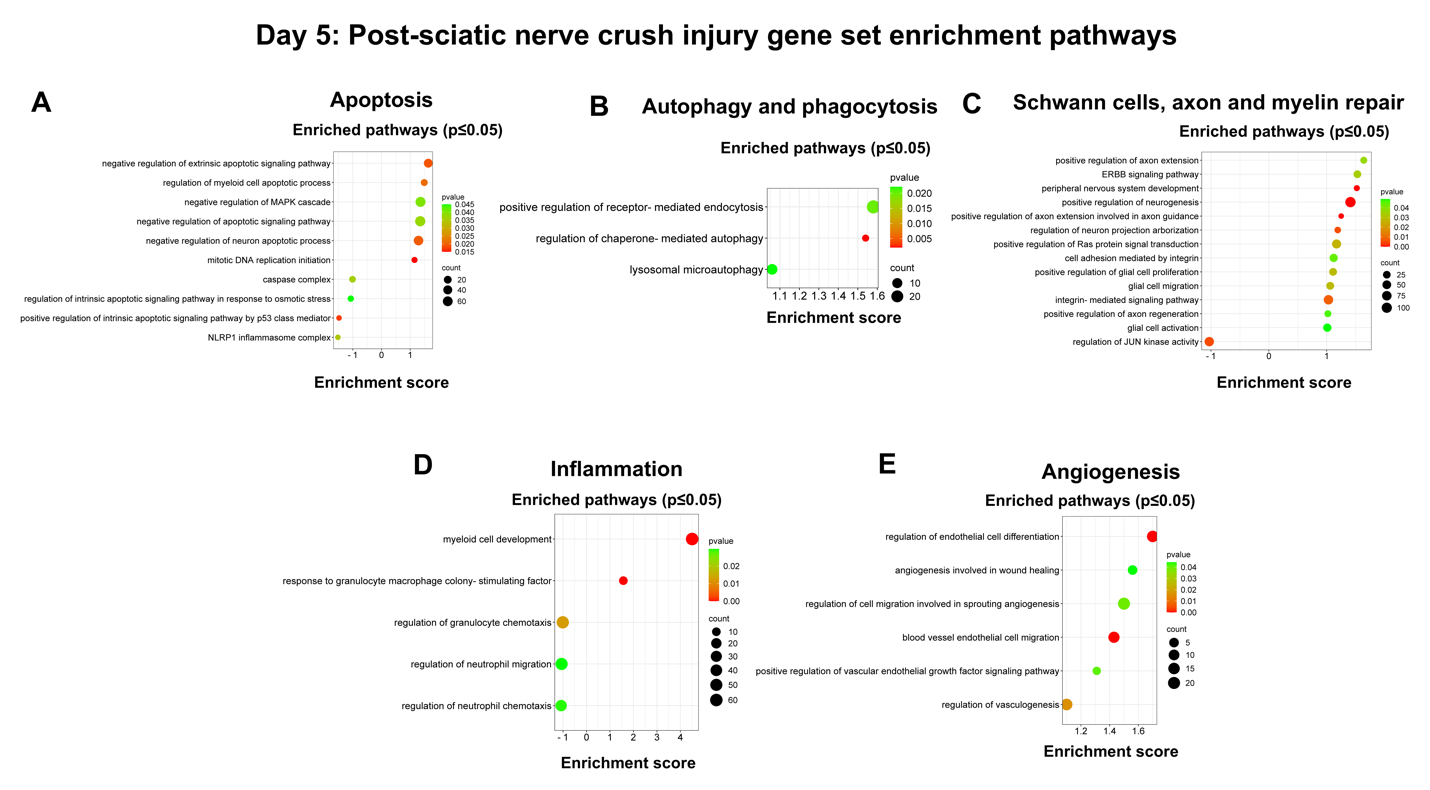
Supplementary Fig. 2: On Day 5, bulk RNA sequencing revealed EPO-enriched genes for biological pathways in nerves following SNCI. A-E** Gene set enrichment assay (GSEA) pathways are represented on the y-axis with their associated gene numbers (p ≤ 0.05), while the x-axis displays the enrichment score for each pathway. Saline vs. EPO treatment, n = 4/ group.

**
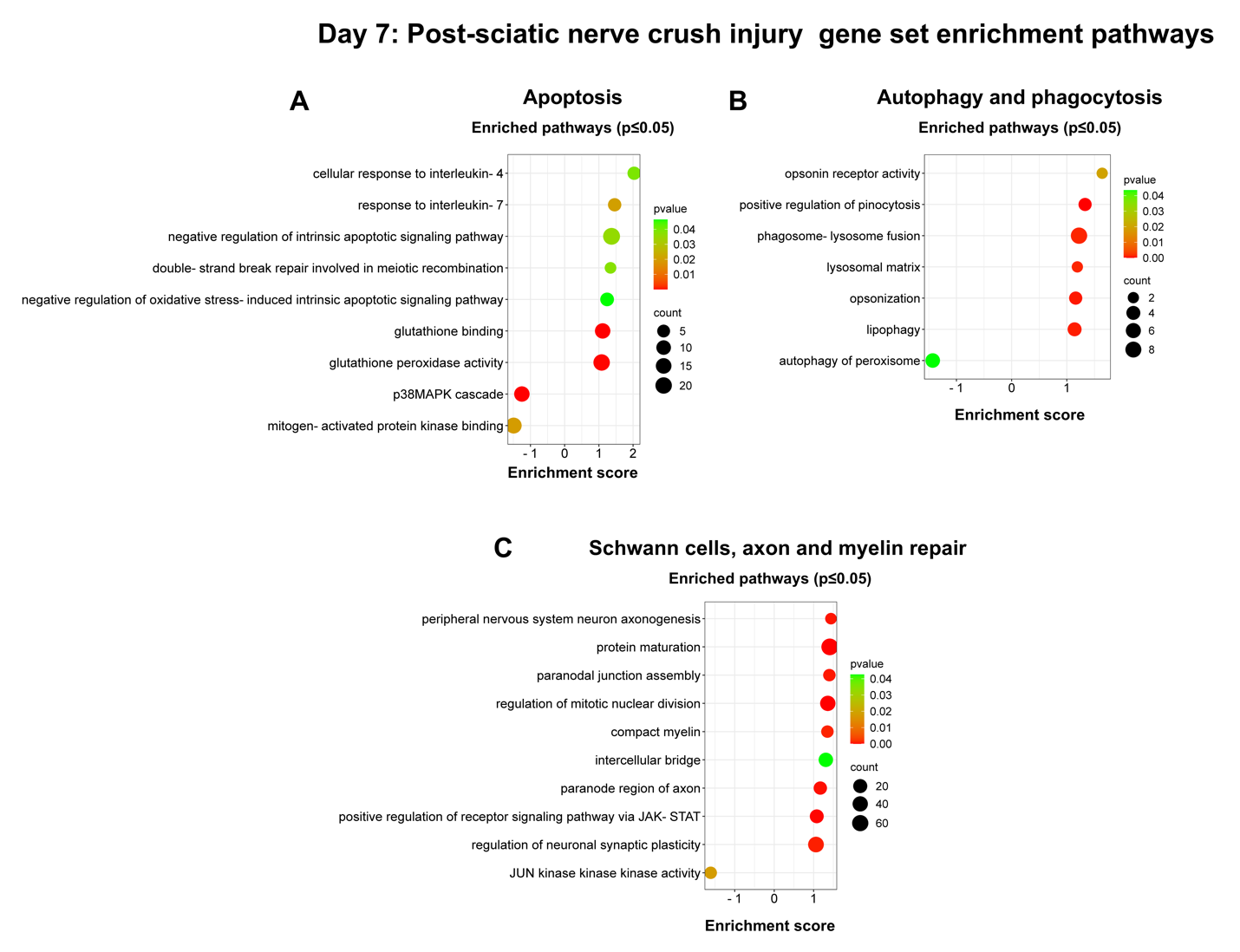
Supplementary Fig. 3: On Day 7, bulk RNA sequencing revealed EPO-enriched genes for biological pathways in nerves following SNCI. A-E** Gene set enrichment assay (GSEA) pathways are represented on the y-axis with their associated gene numbers (p ≤ 0.05), while the x-axis displays the enrichment score for each pathway. Saline vs. EPO treatment, n = 4/ group.

**
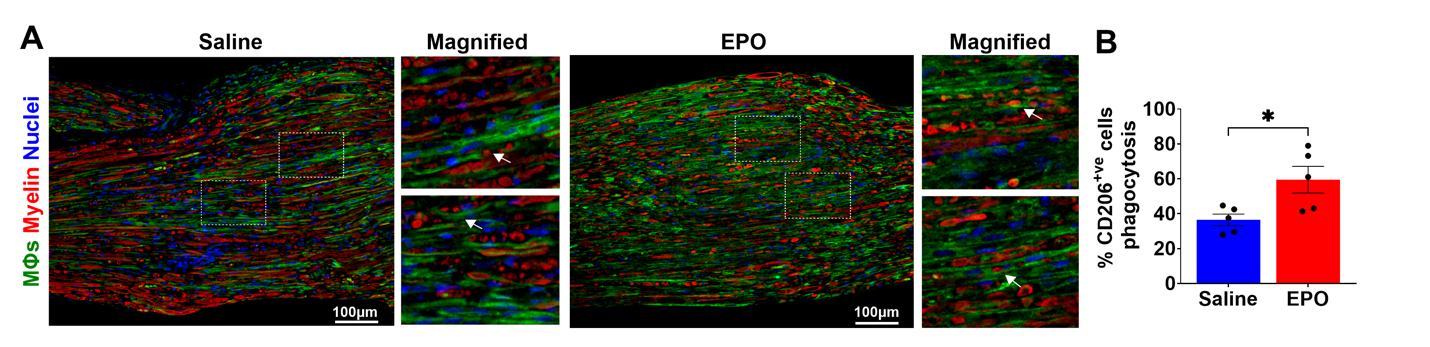
Supplementary Fig. 4: A, B** Representative IHC images and quantitative results of M2 macrophages (anti-CD206 staining) phagocytosis of myelin debris (anti-MPZ staining) in saline and EPO treated nerve tissues on post-SNCI day 5. n = 5/ group. Data are represented as mean ± SEM. The statistical significance is indicated by asterisks (*P < 0.05 vs. saline group) and compared using two-tailed, unpaired t-tests.

**
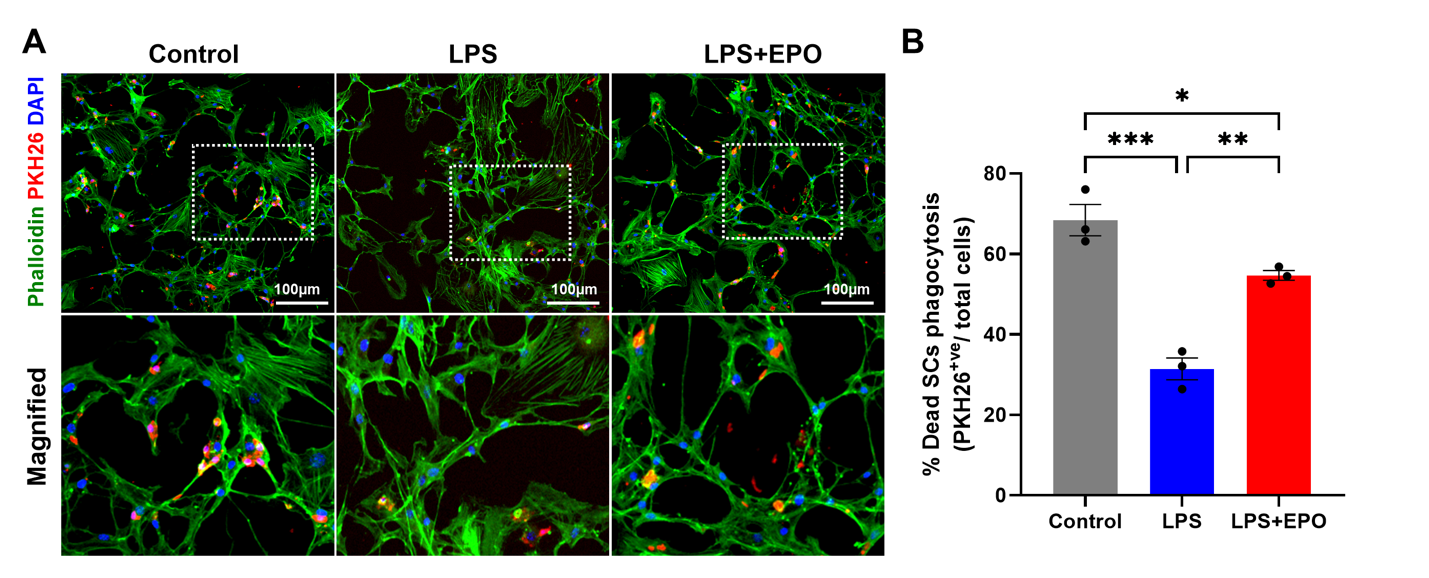
Supplementary Fig. 5: A, B** Representative IF images and quantitative results of repair SCs (phalloidin staining) phagocytosis of myelin debris (PKH26 staining) following 24h EPO (10IU/ mL) treatment under LPS (500ng/ mL) stress conditions. n = 3/ group. Data are represented as mean ± SEM. The statistical significance is indicated by asterisks (*P < 0.05, **P <  0.0021, and ***P < 0.0002 vs. saline group) and compared using ordinary one-way ANOVA.
