# Supplementary figures and images for: Erythropoietin decreases apoptosis and promotes Schwann cell repair and phagocytosis following nerve crush injury in mice
Source: Cell Death Dis. 2025 Jul 3;16(1):490. doi: 10.1038/s41419-025-07825-4 (PMC12229349; doi:10.1038/s41419-025-07825-4)

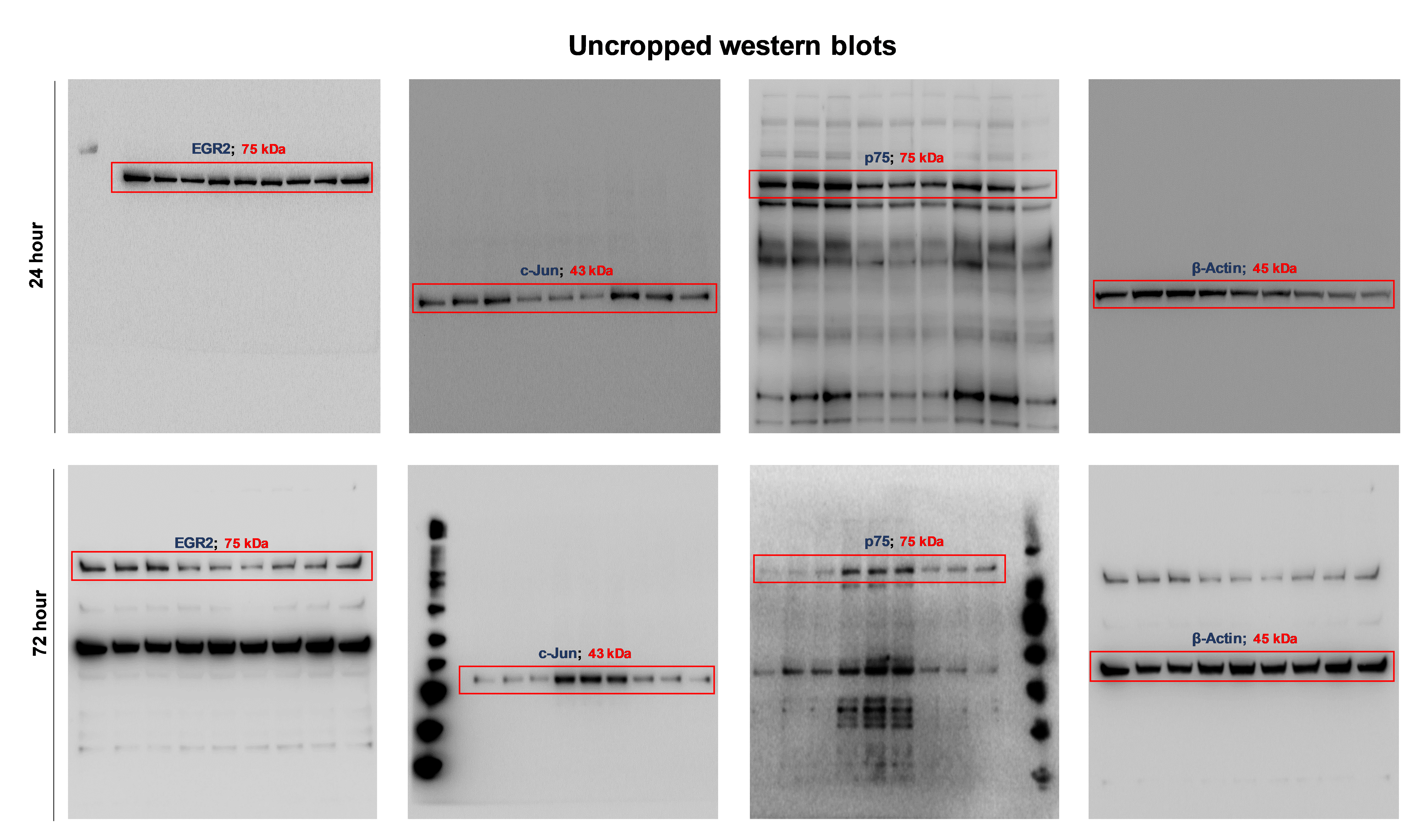

Supplement: Supplementary file 2 — Supplemental Material [file 41419_2025_7825_MOESM2_ESM.png]
