# Supplementary material for: Erythropoietin decreases apoptosis and promotes Schwann cell repair and phagocytosis following nerve crush injury in mice
Source: Cell Death Dis. 2025 Jul 3;16(1):490. doi: 10.1038/s41419-025-07825-4 (PMC12229349; doi:10.1038/s41419-025-07825-4)
Supplement: Supplementary file 9 — Supplementary_Table 7 [file 41419_2025_7825_MOESM9_ESM.docx]

| **List of materials and sources** |  |  |
| --- | --- | --- |
| **Chemical Reagents** | **Source** | **Catalogue number** |
| Xylazine | Dechra Veterinary Products | N/A |
| Ketamine hydrochloride | Dechra Veterinary Products | N/A |
| Betadine Solution | Dailymed | NDC67618-155-16 |
| Buprenorphine | Fidelis Animal Health | NDC86084-100-30 |
| Epoetin | Retacrit | NDC 0069-1305-10 |
| Trizol | Invitrogen | 15596026 |
| RNA isolation kit | Thermo Fisher Scientific | 12183016 |
| DNA HS reagent | Thermo Fisher Scientific | Q32854 |
| DAB-TUNNEL kit | Abcam | ab206386 |
| ProLong^TM^ Gold DAPI | Thermo Fisher Scientific | P36935 |
| Protease and phosphatase inhibitor | Thermo Fisher Scientific | 78442 |
| BCA assay kit | Thermo Fisher Scientific | 23225 |
| Schwann cells complete media | ScienCell | 1701 |
| PKH-26 dye | Sigma Aldrich | MIDI26-1KT |
| Flash phalloidin green 488 | BioLegend | 42420 |
| Flow cytometry staining buffer | R&D | FC001 |
| 4% paraformaldehyde | Thermo Fisher Scientific | J19943.K2 |
| RIPA lysis buffer | Sigma Aldrich | R0278 |
| ECL kit | Thermo Fisher Scientific | 34579 |
| **Primary Antibodies** | | |
| p75 | Millipore Sigma | ab1554 |
| MPZ | Aves labs | PZO |
| Ki67 | Cell Signaling Technology | 9129 |
| c-Jun | Santa Cruz | Sc74543 |
| EGR2 | Abcam | ab245228 |
| β-Actin | Cell Signaling Technology | 5125S |
| Conjugated p75 | Bios | BS-0161R |
| **Secondary Antibodies** | | |
| Anti-rabbit Alexa fluor 488 | Invitrogen | A11034 |
| Anti-mouse Alexa fluor 488 | Invitrogen | A32723 |
| Anti-chicken Alexa fluor 647 | Invitrogen | A21449 |
| Anti-rabbit Alexa fluor 647 | Invitrogen | A32733 |
| Anti-rabbit HRP | Cell Signaling Technology | 7074 |
| Anti-mouse HRP | Cell Signaling Technology | 7076 |
